# Association of short-term exposure to air pollutants with mortality from respiratory diseases: a case-crossover study of individual cases based in Hefei, China

**DOI:** 10.7189/jogh.15.04196

**Published:** 2025-06-27

**Authors:** Xiaofeng Lu, Xu Zhang, Tao Zhang, Jianping Ni, Yongzhen Peng, Xuyang Chen, Hanqing Wu, Guosheng Wang, Xiaoyun Fan, Faming Pan

**Affiliations:** 1 Department of Epidemiology and Biostatistics, School of Public Health, Anhui Medical University, Hefei, China; 2 The Inflammation and Immune Mediated Diseases Laboratory of Anhui Province, Anhui Medical University, Hefei, China; 3 Department of Hospital Management Research, The First Affiliated Hospital of Anhui Medical University, Hefei, China; 4 The Department of Geriatric Respiratory and Critical Care Medicine, The First Affiliated Hospital of Anhui Medical University, Hefei, China

## Abstract

**Background:**

Although short-term exposure to air pollutants has been linked to heightened hospital admissions for respiratory diseases (RDs), evidence regarding its association with the risk of mortality from such diseases remains scarce. We aimed to examine the impact of short-term exposure to air pollutants on RD-related mortality.

**Methods:**

We employed a time-stratified case-crossover design to explore the impact of short-term exposure to various air pollutants (fine particulate matter (PM_2.5_), coarse particulate matter (PM_10_), nitrogen dioxide (NO2), sulfur dioxide (SO_2_), carbon monoxide (CO), and ozone (O_3_)) on mortality due to RDs. Our sample comprised 15 878 RD-related deaths that occurred between 2017 and 2020 in Hefei, Anhui province. We delineated daily exposure to air pollutants using raster data corresponding to the residential addresses of the study subjects. We used conditional logistic regression to assess the relationship between pollutant exposure and the risk of mortality. To improve precision in identifying vulnerable populations, we stratified individuals by gender and age. Lastly, we used interrupted time series (ITS) analysis to explore COVID-19's impact on RD mortality.

**Results:**

We found that every 10 μg/m3 increase in PM_10_, PM_2.5_, and CO at a lag of zero days was associated with the largest significant effect on increased mortality from RDs (excess mortality risks of 0.735%, 1.349%, and 0.160%, respectively). We observed the highest positive associations between NO_2_ and O_3_ with delays of one and two days, resulting in excess mortality risks of 1.965% and 0.861%, respectively. We obtained consistent findings through moving average concentration analysis, while our stratified analysis showed that females and the elderly exhibited a heightened susceptibility to mortality from RDs attributable to short-term exposure to pollutants. Additionally, the ITS analysis confirmed that the COVID-19 outbreak did not significantly alter the level and trend of RD deaths.

**Conclusions:**

Our findings indicate that short-term exposure to air pollutants, excluding SO_2_, increases mortality rates from RDs, particularly among women and the elderly. In the context of Hefei, our findings highlight the public health imperative for reducing local residents' exposure to air pollutants, particularly through targeted protection of vulnerable populations, to minimise preventable RD mortality.

Respiratory diseases (RDs) primarily affect the lungs, chest, trachea, and bronchi in the human body. Mild cases often present with symptoms such as coughing, chest pain, and tightness, while severe cases may progress to dyspnea and respiratory failure, ultimately resulting in death [1]. Chronic RDs impose a significant global health burden, contributing substantially to morbidity and mortality rates, even being ranked as the third leading cause of death in 2017 [2]. This was paralleled by increases in fossil fuel use and industrialisation that have led to air pollution, further elevating public health risks related to RDs [3]. This is especially concerning for human health, as research has shown that particulate matter, particularly fine particulate matter (PM_2.5_) and coarse particulate matter (PM_10_), has been linked to a growing burden of premature mortality [4]. Similarly, epidemiological studies have suggested a strong association between poor air quality and increased morbidity and mortality from respiratory and cardiovascular diseases [5–7].

Anhui Province, where Hefei is located, has historically been an industry-dominated region, with manufacturing sectors (*e.g.* electrical equipment, coal processing) experiencing rapid development during the early 2010s. As one of China's more polluted cities [8] which has also been experiencing rapid economic growth, Hefei now faces the challenge of balancing development priorities with public health protection.

Air pollutants can impair respiratory immune function [9], with sulfur dioxide (SO_2_) and nitrogen dioxide (NO_2_) being known to induce acute stress and inflammation in bronchial epithelial cells. Besides this, PM_2.5_ exposure has been found to have delayed adverse effects, potentially linked to deaths from lung cancer, stroke, and other diseases [10,11]. Researchers have been committed to exploring the impact of environmental exposure on RD mortality. An epidemiological inquiry into RD mortality and hospital admissions across southeast China delineated regional disparities in the uptick of RD mortality attributable to air pollutant exposure [12]. In Hangzhou, the authors noted robust correlations between PM_2.5_, NO_2_, and SO_2_ levels and RD fatalities, while in Zhoushan City, significant effects were only evident for PM_2.5_ and ozone (O_3_) [12]. This makes it important to further study the changes in air pollutant exposure and RD mortality in different regions.

Previous studies examining the impact of air pollution on RDs have primarily focussed on outpatient visits among patients afflicted with RDs [13–15]. However, there remains a gap in research regarding the influence of air pollutant exposure on mortality associated with RDs. The time series analysis conducted by Shao and colleagues in Hefei City revealed significant correlations between increased concentrations of NO_2_ and O_3_ and heightened risks of RD mortality [16]. However, the authors observed no significant associations between PM_10_ and SO_2_ concentrations and RD mortality. This discrepancy may partly stem from the utilisation of pollutant data sourced from monitoring sites, which may lack precision in assessing individual exposure levels. Furthermore, the investigation did not examine the impact of PM_2.5_ exposure on the percentage change in RD mortality. Similarly, O_3_ and carbon monoxide (CO) were not considered adequately in prior research, and the reported effects of other pollutants on RDs varied across studies, highlighting the critical need for additional investigation.

Here we investigated the impact of air pollutant exposure on RD-related mortality among the entire population of Hefei, China, and conducted stratified analyses to identify susceptible subpopulations. To mitigate potential confounding factors, we implemented case-crossover design, utilising individuals as their own controls. We hope our findings will offer a robust theoretical foundation for informing the development of public health interventions and policies aimed at addressing climate and environmental changes in the future.

## METHODS

### Study area

Hefei, situated in the Yangtze River Delta, is the capital city of Anhui Province, China (Figure S1 in the **Online Supplementary Document**). It has a total area of 11 445.1 km^2^ and a permanent population above 8.18 million. Positioned at 31°49'N, 117°13'E, the city experiences distinct seasonal variations, attributable to its unique geographical location within the subtropical humid monsoon zone. It is one of China’s four major science and technology centres, and has thus experienced rapid economic growth in recent years. However, alongside economic progress, the city has also seen a parallel decline in air quality.

### Death data

We obtained our data from the death record system of the Hefei Center for Disease Prevention and Control, which recorded all causes of death data in the region. Our specific subset of data spanned the period from 1 January 2017 to 31 December 2020. We screened RD deaths according to the International Classification of Diseases, 10th revision (ICD-10) codes to obtain the daily number of deaths (J00–J99). For each subject, we extracted information including date of death, gender, age, place of residence, and diagnostic criteria.

### Meteorological and pollutant data

We retrieved our air pollution data from the ChinaHighAirPollutants database [17], which contains the daily air pollutant data set of China, providing complete spatial and temporal environmental coverage of the country, with spatial accuracy ranges for PM_2.5_ and PM_10_ at 1 km × 1 km and for SO_2_, NO_2_, CO, and O_3_ at 10 km × 10 km. The meteorological data comes from the Hefei Meteorological Bureau, including daily average temperature (°C) and relative humidity (%).

### Research design

We used a time-stratified case-crossover design to investigate the correlation between exposure to air pollutants and mortality from RDs. As this is a type of observational study, we report our findings per the STROBE guidelines (Checklist S1 in the **Online Supplementary Document**) [18].

The case-crossover design is used for assessing both short-term and long-term impacts of environmental exposure on human health, offering a mechanism to mitigate potential confounding variables [19,20]. Specifically, our analysis considered the day of death and the environmental exposure before and after the day of death (month of death) of the study subjects, with each subject being used as a reference for themselves. The death date of the subject is defined as the case day, with the same month but different weeks as the control day (for example, 15 May is the case day, while 8 May, 1 May, 22 May, and 29 May are the control days). 

Our study leans on prior research which demonstrated that short-term effects of air pollutants on RD mortality and morbidity remain an active research focus [21]. Previous investigations of RD mortality predominantly employed relatively short lag periods [22,23]. Given the acute exacerbations and high mortality risk of RDs, we selected a lag period of 0–3 days for analysis. We geocoded each included participant by their address to obtain air pollutant concentration values (the 24-hour average concentration of PM_2.5_, SO_2_, NO_2_, CO, and PM_10_, and the maximum 8-hour average O_3_ concentration per day) on the day of the case and the day of the control.

As our data spanned the period 1 January 2017 to 31 December 2020, and as the COVID-19 outbreak started in China in early 2020, we used interrupted time series (ITS) analysis to explore whether the pandemic impacted RD mortality. The ITS is widely used in the evaluation of public health policies and measures [24–26], while in epidemiological studies, scholars have used the approach to analyse the effects of vaccine use on population health [27]. Here, we treated the COVID-19 outbreak as an ‘intervention’ and used the ITS analysis to identify whether COVID-19 has had a significant impact on RD deaths, per the following formula:

*Yt* = *β*0 + *β*1*T* + *β*2*Xt* + *β*3(*T* − *T*0)*Xt*

*β*0 is the baseline level, *β*1 is the change in outcome associated with each unit increase in time (representing the underlying pre-intervention trend), *β*2 is the level change following the intervention, and *β*3 is the slope change post-intervention (with *T*0 as the time of intervention initiation).

### Statistical analysis

The case-crossover design allows for full control over the basic information of the study subjects (age, lifestyle, and other comorbidities) as well as the impact of seasonality, holidays, and long-term trends during the analysed time period [28,29]. Our main analysis focussed on the impact of short-term exposure to pollutants on RD mortality, *i.e.* examined pollutant concentrations on the day of death and three days preceding it. We employed conditional logistic regression to quantify the association between air pollutants and RD mortality. Pollutants served as the primary exposure variables, while meteorological factors (mean temperature, relative humidity, and diurnal temperature range) were accounted for using natural splines with three degrees of freedom to control for their potential influence. We presented these results as percentage changes in the odds ratios of mortality from RDs and their 95% confidence intervals (95% CIs) for every 10 μg/m3 increase in six air pollutants. To balance the effect of daily dynamic levels of air pollutants on the results, we calculated a moving average of pollutants for the three days before the death date (average of air pollutant concentrations today *vs*. a day ago (lag0_1); today *vs*. yesterday *vs*. two days ago (lag0_2); and today, yesterday, two days, and three days ago (lag0_3)). We assessed the nonlinear relationship between air pollutants and RD mortality and controlled for holiday effects (using a natural spline of three degrees of freedom to adjust for mean temperature, relative humidity, and diurnal temperature range).

To determine whether any populations were especially sensitive to this association, we conducted stratified analyses based on sex and age groups (0–65 years and ≥65 years). We also conducted sensitivity analyses to assess the robustness of our main results, whereby we manipulated the degrees of freedom of the spline function for meteorological factors to observe its impact on the analysis outcomes. For this purpose, we used the Akaike information criterion to evaluate the performance of different model functions [30].

We conducted all analyses in R, version 4.2.3 (R Core Team, Vienna, Austria). A two-sided *P*-value <0.05 denoted statistical significance.

## RESULTS

### Basic data description

In our study sample, 15 878 individuals succumbed to RDs, generating 58 327 control days. Among these fatalities, 61.4% were male and 38.6% were female. There were 13 253 deaths among seniors aged ≥65 years, while 2625 deaths occurred among adults aged <65 years. National holidays contributed to 25.4% of the total number of deaths (Table S1 in the **Online Supplementary Document**).

Over the study period, the average concentrations of PM_2.5_, SO_2_, NO_2_, CO, PM10, and O_3_ were recorded as 49.91 μg/m^3^, 11.41 μg/m^3^, 39.83 μg/m^3^, 858.89 μg/m^3^, 79.86 μg/m^3^, and 101.66 μg/m^3^, respectively. The average temperature was 15.42°C and the daily temperature difference averaged at 9.73°C, while relative humidity was 76.73% (Table S2 in the **Online Supplementary Document**).

### Overall exposure-response relationships

**Figure 1:**
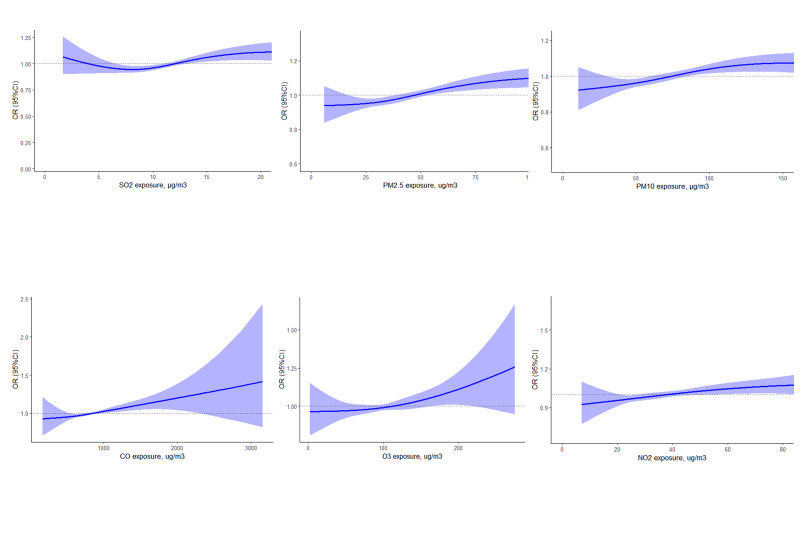
Exposure-response curve between 0–3 days of air pollutant exposure and RD mortality. The solid line is the effect estimate and the shaded part is the 95% CI. CI – confidence interval, OR – odds ratio.

**Figure 2:**
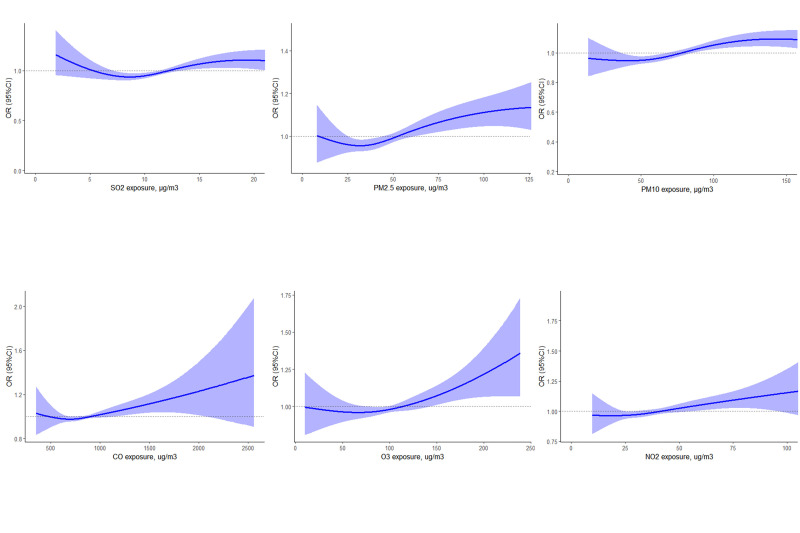
Exposure-response curve between air pollutant moving average concentration exposure and RD mortality at 0_3 days. The solid line is the effect estimate and the shaded part is the 95% CI. CI – confidence interval, OR – odds ratio.

During the exposure period spanning from lag 0 to lag 3 days, the risk of RD mortality increased with increasing concentrations of six air pollutants. Notably, CO and NO_2_ showed a discernible linear correlation trend (**Figure 1**). The results of the sliding average concentration analysis further confirmed the significant effect between pollutant exposure and increased mortality from RDs under conditions that controlled for daily fluctuations in pollutant concentrations (**Figure 2**). This demonstrates an amplification effect of multi-day cumulative exposure, with moving average effects for all pollutants exhibiting stronger associations than single-day exposures.

### Single-day lag effects

Throughout the three-day lag period, exposure to PM_2.5_, PM_10_, CO, and NO_2_ exhibited acute effects, showcasing a positive association with the risk of death from RDs on both day 0 and day 1. The observed excess risk of death ranged from 0.105% to 1.965% for each additional 10 μg/m^3^. Conversely, O_3_ demonstrated significance only with a 2-day lag, where the risk of excess death was recorded at 0.861% (ranging from 0.022% to 1.708%). We found no statistical association between SO_2_ and mortality from RDs (**Table 1**).

**Table 1:** Additional risk of death associated with each 10 μg/m^3^ increase in air pollutants*

	Died of respiratory disease	
**Lag by air pollutant (in days)**	**Mortality (95% CI)**	***P*-value**
**SO_2_**		
0	5.379 (−2.068, 13.391)	0.16
1	1.639 (−5.601, 9.439)	0.67
2	1.849 (−5.409, 9.662)	0.62
3	−5.636 (−12.361, 1.605)	0.12
**PM_2.5_**		
0	1.349 (0.442, 2.265)	0.002
1	1.391 (0.488, 2.301)	0.002
2	0.714 (−0.176, 1.612)	0.12
3	0.686 (−0.188, 1.567)	0.13
**PM_10_**		
0	0.735 (0.113, 1.361)	0.02
1	0.640 (0.021, 1.263)	0.04
2	0.342 (−0.275, 0.963)	0.28
3	0.305 (−0.313, 0.927)	0.33
**CO**		
0	0.160 (0.069, 0.253)	0.007
1	0.105 (0.015, 0.201)	0.02
2	0.054 (−0.036, 0.143)	0.24
3	0.052 (−0.037, 0.141)	0.25
**O_3_**		
0	0.350 (−0.476, 1.183)	0.06
1	0.811 (−0.028, 1.656)	0.06
2	0.861 (0.022, 1.708)	0.04
3	0.636 (−0.209, 1.488)	0.14
**NO_2_**		
0	1.797 (0.286, 3.332)	0.02
1	1.965 (0.439, 3.514)	0.01
2	1.422 (−0.085, 2.951)	0.06
3	−0.001 (−1.501, 1.521)	0.66

CI – confidence interval

*Lag 0 = day of death. Lag1 = one day before day of death. Lag2 = two days before day of death. Lag3 = three days before day of death.

### Moving average effects

In the lag of 0_1 day, CO showed the largest positive correlation effect, with an excess mortality risk of 0.166% (95% confidence interval (CI) = 0.066, 0.267). Meanwhile, NO_2_ and PM_10_ had the strongest association with an increased risk of death from RD within a lag of 0_2 days, with excess mortality risks of 2.479% (95% CI = 0.687, 4.303) and 0.925% (95% CI = 0.175, 1.681), respectively. Furthermore, PM_2.5_ and O_3_ showed the greatest positive correlation effect with a lag of 0_3 days, with risks of excess mortality at 1.836% (95% CI = 0.672, 3.013) and 1.538% (95% CI = 0.392, 2.698), respectively. We likewise did not find a statistical association between the moving average concentration of SO_2_ and RD mortality (**Table 2**).

**Table 2:** Additional risk of death associated with each 10 μg/m^3^ increase in air pollutants*

	Died of respiratory disease		
**Lag by air pollutant (in days)**	**Mortality (95% CI)**	***P*-value**	
**SO_2_**			
0_1	4.280 (−4.103, 13.394)	0.32	
0_2	4.612 (−4.524, 14.623)	0.33	
0_3	0.650 (−8.717, 10.978)	0.89	
**PM_2.5_**			
0_1	1.770 (0.761, 2.790)	0.001	
0_2	1.810 (0.721, 2.912)	0.001	
0_3	1.836 (0.672, 3.013)	0.003	
**PM_10_**			
0_1	0.886 (0.201, 1.580)	0.01	
0_2	0.925 (0.175, 1.681)	0.02	
0_3	0.919 (0.111, 1.733)	0.03	
**CO**			
0_1	0.166 (0.066, 0.267)	0.003	
0_2	0.158 (0.051, 0.265)	0.004	
0_3	0.150 (0.037, 0.264)	0.006	
**O_3_**			
0_1	0.830 (−0.124, 1.792)	0.08	
0_2	1.283 (0.229, 2.347)	0.02	
0_3	1.538 (0.392, 2.698)	0.006	
**NO_2_**			
0_1	2.245 (0.565, 3.952)	0.01	
0_2	2.479 (0.687, 4.303)	0.01	
0_3	2.004 (0.119, 3.923)	0.04	

CI – confidence interval

*Lag0_1 = mean concentration between day of death and one day prior. Lag0_2 = mean concentration of day of death and one and two days prior. Lag0_3 = mean concentration of day of death and one, two, and three days prior.

### Pollutant-specific risk ranking

**Figure 3:**
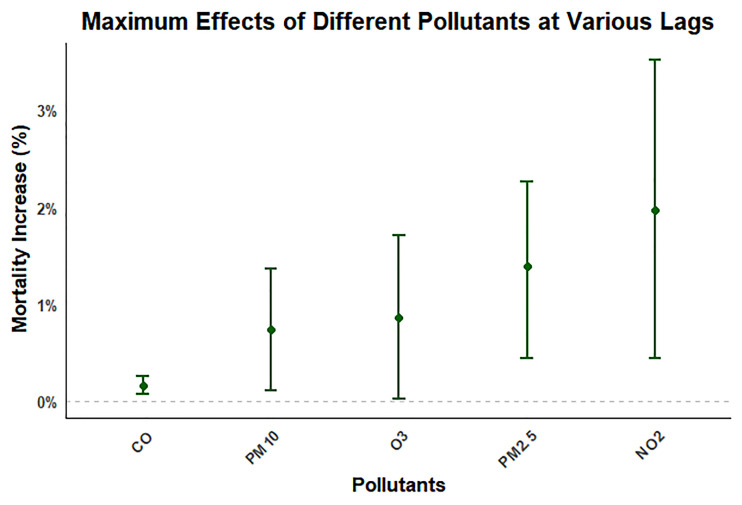
The maximum effective effect size of the percentage increase in RD mortality for every 10 μg/m^3^ increase in air pollutant exposure at different lag days. CI – confidence interval, OR – odds ratio.

Through analysing the maximum effects of exposure to different pollutants on the percentage change in RD mortality during a three-day lag period (**Figure 3**), we found that short-term exposure to NO_2_ led to the highest risk of increasing RD death among all pollutants, followed by PM_2.5_, O_3_, PM_10_, and (lastly) CO. This indicates that there are some differences in the acute effects of short-term exposure of different pollutants on RD mortality. Moving average concentrations also show similar analysis results (Figure S2 in the **Online Supplementary Document**). When we exclude the effect of short-term fluctuations in air pollutants is excluded, the percentage increase in mortality from RDs caused by pollutant exposure was higher. Consistent with the analysis results of different lag days, NO_2_ and CO still contributed the most and least to the increase of RD mortality.

### Stratified analyses

Stratified analyses based on sex and age showed some variation in the association between pollutants and a percentage increase in RD mortality in different populations. Specifically, PM_2.5_, PM_10_, CO, and NO_2_ were significantly associated with increased RD mortality in women and the elderly people aged ≥65 years, while we found no statistical association for adults aged <65 years and men. The pollutant with the strongest correlation was NO_2_, with an excess mortality risk of 5.438% (95% CI = 2.503, 8.458) and 2.455% (95% CI = 0.490, 4.458) for RD. Excluding the male group, O_3_ showed a positive correlation with the female group and the age-stratified group. The percentage increase in RD mortality in the female population was 2.765% (95% CI = 0.96, 4.657) for every 10μg/m3 increase in O3 exposure, and the significant effect was 2.125% (95% CI = 0.96, 4.657) for young and old people, respectively. 0.075, 4.216), 1.437% (95% CI = 0.185, 2.705), and adults aged <65 years seem to be more susceptible to the risk effects of O_3_ exposure than elderly people aged ≥65 years (**Table 3**). We otherwise found a correlation between the percentage change in SO_2_ and RD mortality rate in stratified analysis.

**Table 3:** Relationship between exposure to air pollutants and mortality rate, stratified by gender and age*

	Died of respiratory disease	
**Variables, stratified by pollutant**	**Mortality (95% CI)**	***P*-value**
**SO_2_**		
Sex		
*Male*	1.548 (−7.563, 11.556)	0.75
*Female*	14.546 (−0.789, 32.252)	0.06
Age in years		
*<65*	9.245 (−13.981, 38.754)	0.46
*≥65*	6.857 (−1.373, 15.774)	0.1
**PM_2.5_**		
Sex		
*Male*	0.836 (−0.282, 1.966)	0.14
*Female*	4.011 (2.369, 5.679)	0.004
Age in years		
*<65*	0.991 (−1.256, 3.289)	0.38
*≥65*	2.138 (0.945, 3.345)	0.002
**PM_10_**		
Sex		
*Male*	0.356 (−0.430, 1.148)	0.38
*Female*	2.155 (1.051, 3.271)	0.008
Age in years		
*<65*	0.598 (−0.941, 2.159)	0.45
*≥65*	1.139 (0.318, 1.967)	0.01
**CO**		
Sex		
*Male*	0.084 (−0.033, 0.200)	0.16
*Female*	0.333 (0.174, 0.491)	0.003
Age in years		
*<65*	0.154 (−0.067, 0.374)	0.17
*≥65*	0.188 (0.079, 0.298)	0.003
**O_3_**		
Sex		
*Male*	0.757 (−0.582, 2.114)	0.27
*Female*	2.765 (0.906, 4.657)	0.03
Age in years		
*<65*	2.125 (0.075, 4.216)	0.04
*≥65*	1.437 (0.185, 2.705)	0.02
**NO_2_**		
Sex		
*Male*	0.667 (−1.445, 2.823)	0.54
*Female*	5.438 (2.503, 8.458)	0.007
Age in years		
*<65*	2.869 (−0.855, 6.732)	0.13
*≥65*	2.455 (0.490, 4.458)	0.01

CI – confidence interval

### ITS analysis

The ITS analysis showed that the baseline level of RD deaths before the onset of COVID-19 was 615.52. The estimated impact of each time unit change on RD deaths was −13.45. The estimated impact of COVID-19 on the level of RD deaths was 208.67 (*P* = 0.3), but this result lacked significance. Similarly, COVID-19 did not have a statistically significant effect on the change in the time trend of RD deaths (estimated impact = −0.9813, *P* = 0.88). Based on the results of ITS analysis, we observed that time had a significant impact on RD death, and with the passage of time, the death of RD was significantly reduced (Figure S3 in the **Online Supplementary Document**). In addition, the death toll of RD had a trend of decreasing first and then increasing in one year. However, the outbreak did not have a significant effect on the level and trend of RD deaths. In other words, the outbreak interventions did not significantly change the level and trend of RD deaths.

## DISCUSSION

Based on a population in Hefei, China, we investigated the association between RD mortality and exposure to air pollutants from 2017 to 2020, employing a case-crossover design and encompassing a total of 15 878 RD deaths. The analysis showed a significant correlation between short-term (0–3 days) exposure to every 10 μg/m^3^ increase in air pollutants and heightened RD mortality, with the exception of SO_2_. We also conducted a stratified analysis based on gender and age, which showed that women and older people were more vulnerable to pollutants. The exposure-response curve presented a linear correlation between air pollutant exposure and RD mortality risk, with varying degrees of correlation intensity observed across different pollutants.

To the best of our knowledge, this study is the first to use a case-crossover study to explore the association between short-term pollutant exposure and percentage changes in RD mortality in Anhui Province. In it, we quantified the increased mortality from RD caused by exposure to air pollutants and identified vulnerable populations, thus contributing evidence to inform healthcare professionals in addressing the challenges that climate change poses to people with RD. The consistent significance of pollutant exposure during acute episodes suggests that immediate protective interventions for high-risk populations may yield maximal health benefits and that the health effects of short-term exposure to pollutants (except SO2) intensify with prolonged exposure duration, necessitating sustained environmental monitoring and pre-emptive implementation of protective interventions for susceptible populations. We note that prior investigations into the impact of air pollutant exposure on RD risk have yielded inconsistent findings. For instance, one study documented a 10.10% surge in the risk of hospital admissions for RD with every 10 μg/m^3^ rise in daily SO_2_ exposure [31], while another observed a cumulative lag effect of SO_2_ on RD mortality [32]. However, other studies found no significant association between SO_2_ exposure and respiratory ailments in children [33] or emergency department hospitalisations for RD [34]. We similarly found no correlation between SO_2_ exposure and RD mortality. Discrepancies in the outcomes of studies on SO_2_ and RD mortality risk may stem from variations in the spatial distribution of air pollutant concentrations. Another potentially important reason is that the SO_2_ concentration in this study (95th percentile of 19.62 µg/m^3^) was lower than the SO_2_ levels published by the WHO for changes in human lung function and respiratory symptoms 24-hour average concentration (40 µg/m^3^) [35]. Future studies should investigate the SO_2_ exposure-RD mortality association across multiple representative regions to further elucidate the health impacts of SO_2_ exposure.

Studies have consistently shown that elevated concentrations of PM_2.5_ and PM_10_ are associated with increased RD mortality [36,37]. Short-term exposure to particulate matter is significantly associated with higher RD mortality, though this effect diminishes with longer exposure windows, suggesting an acute impact [38]. Here we found that an increase in RD mortality during a 0–3-day lag period was associated with a 10 μg/m3 increase in PM_2.5_ and PM_10_ exposure on the day of death and the day before death, which is consistent with previous findings [37,38]. Multi-regional investigations have further underscored regional disparities in the impact of particulate matter on disease morbidity and mortality [39–41]. These regional variations could be attributed, in part, to differences in ethnic composition, genetic susceptibility, proportion of the elderly population, and environmental factors such as temperature and humidity [42–44]. Moreover, research suggests that the chemical constituents of particulate matter exacerbate its adverse health effects, triggering oxidative stress, inflammation, and iron sequestration [45–47]. We speculate that the components of particulate matter can also irritate the body and produce toxicity to the respiratory system, which can lead to the death. Our moving average concentration analysis further confirmed a significant association between PM_2.5_ and PM_10_ exposure and increased RD mortality (0.886% to 1.836%). These findings highlight the need for RD patients to minimise daily exposure to particulate matter to reduce health risks.

Various studies have presented conflicting findings regarding the impact of CO on RD. Studies conducted in Dongguan, Wuhan, Yichang, and Lanzhou in China showed that CO exposure increased the risk of RD incidence by 20.07%, 13%, 21.79%, and 21.6%, respectively [48–51]. Conversely, surveys conducted in Shanghai and Hong Kong, China, did not discern a significant effect of CO exposure on the overall incidence of RDs [52,53]. Regional differences of CO on RD risk can be explained by regional differences in CO concentration and individual susceptibility. Nevertheless, there remains a paucity of data on the relationship between CO exposure and RD mortality risk: here, our expose-response curve shows a near-linear effect of CO on RD mortality, highlighting its significant harm and the need for vigilance against its mortality risks.

For O_3_, a study involving 372 cities showed that with a lag of 0–1 day, the total mortality risk ranges from 0.04% to 0.29% for every 10 μg/m^3^ increase in O_3_ concentration, indicating that the effect of O_3_ increases with the increase of exposure time [54]. Additionally, the synergistic effect of O_3_ and PM_2.5_ was substantial, with exposure to elevated concentrations of both pollutants resulting in significantly higher mortality rates compared to exposure to each pollutant alone [54]. Our findings align with previous research [54]. During the 0–3-day exposure window period, we only found a significant association between O_3_ and the increase of RD mortality after a delay of two days. We found no correlation in other lag periods, which may be due to competition among pollutants. Further, O_3_ contribution to RD mortality was weaker than that of other air pollutants in other lag periods except the second day, meaning that under O_3_ exposure conditions with a two-day lag, the effect of O_3_ on the risk of RD death was not masked by the effect levels of other pollutants. The cumulative effects of O_3_ exposure were assessed using moving averages of O_3_ concentrations over three days. We found that the significant increase in the percentage of RD mortality caused by an increase of 10 μg/m3 of moving average concentration of O_3_ appeared in the lag period of 0_2 and 0_3 days. Due to the rapid economic growth, Hefei City is faced with continuous O_3_ pollution [55]. For this reason, our findings provide a scientific foundation for policymakers in Hefei, a hub of economic and technological advancement, to tackle the health challenges posed by O_3_ pollution.

A nationwide analysis found that a 10 μg/m^3^ increase in NO_2_ concentration raised RD mortality by 1.4%, with higher risks in southern China (1.7%) compared to northern regions (0.7%) [56]. Hefei City, situated in the Yangtze River Delta region, is a representative city for the region of southern China. Our findings closely align with those of a previous study [56], indicating that with a lag of 0–1 day, the mortality rate increased by 1.797% and 1.965% for every 10 μg/m^3^ rise in NO_2_ concentration. Results from a study conducted in Asia on public health and air pollution found a similar trend, whereby every 10 μg/m^3^ increase in NO_2_ concentration was associated a 1.63% rise in RD mortality in Bangkok in Thailand and Shanghai, Hong Kong, and Wuhan, China [57]. On the whole, these findings highlight then need to address the risks posed by NO_2_ exposure in relation to RD mortality.

In our subgroup analysis, we found that exposure to PM_2.5_, PM_10_, O_3_, CO, and NO_2_ was statistically associated with increased RD mortality in the elderly and female populations, while O_3_ was also significantly associated with increased RD mortality in young people. According to prior reports, there is a higher proportion of elderly individuals in southern Chinese provinces compared to northern regions – a geographical difference which likely contributes to a greater risk of increased RD mortality attributed to NO_2_ exposure in southern China compared to northern regions [58,59]. Compared with young people, the elderly are more likely to suffer from severe RD diseases such as respiratory infections and even respiratory failure due to the degeneration of the body's respiratory function [60]. The proportion of elderly people aged ≥65 years in this study (83.5%) may be one of the reasons for the age stratification analysis results. A study conducted in northeast China showed that increased concentrations of NO_2_ and CO were associated with the development of RD diseases such as asthma, pulmonary infection, and chronic obstructive pulmonary disease in women, while particulate matter was more closely associated with the development of RD in the elderly population [61]. This underscores the adverse impact of air pollutant exposure on RD mortality among women and elderly individuals.

Research on the relationship between lung function and O_3_ exposure in young individuals suggests that higher environmental O_3_ levels can impair airway function, rendering this population more susceptible to the adverse effects of O_3_ exposure, thus explaining the observed increase in RD mortality associated with O_3_ exposure in young people [62]. Female and elderly populations maintain their outdoor activity patterns (*e.g.* shopping, walking) regardless of severe air pollution levels, showing no behavioural modification in response to environmental exposure. In contrast, male populations exhibit different behavioural patterns, potentially resulting in greater pollution exposure impacts on females and elderly individuals [63]. Furthermore, physiological vulnerability, particularly in respiratory pathology [64], is more pronounced in females and elderly populations compared to males. This leads to diminished homeostatic regulatory capacity against pollutant exposure, making females more susceptible to pollution-associated RD mortality risks.

Our study data cover the period from 2017 to 2020, including the outbreak of the COVID-19 pandemic in 2020. Importantly, COVID-19 primarily affects the respiratory system and may cause damage to lungs and airways. Previous studies have reported inconsistent effects of the pandemic on RD mortality and incidence [65–67]. We employed an ITS analysis to control for potential COVID-19 confounding in our results and found no significant changes in RD mortality levels or trends during the pandemic. This could be due to the fact that the Hefei Center for Disease Control implemented containment measures early in the outbreak [68], potentially preventing large-scale community transmission [69]. Therefore, any impacts of the pandemic on our findings can be reasonably excluded.

To the best of our knowledge, this study is the first to fully investigate the relationship between air pollutant exposure and RD mortality in Hefei using a case-crossover design combined with a raster data structure. Diverging from previous methodologies, the case-crossover design enabled us to account for confounding biases arising from individual characteristics, improving the rigour and credibility of our statistical analyses. When we acquire air pollutant data, we use high-resolution raster data, combined with the geographical location coding of the subject, to comprehensively evaluate the impact of pollutants on the subject. Nonetheless, our study has certain limitations. As it was confined to Hefei City, it cannot be generalised to other regions. Although we utilised raster data to evaluate pollutant exposure, we also may not have fully captured individuals’ actual exposure levels. Future studies should account for these factors.

## CONCLUSIONS

We found that short-term exposure to PM_2.5_, PM_10_, CO, O_3_, and NO_2_ was significantly associated with increased percentage of RD mortality. For every 10 μg/m^3^ increase in pollutants during 0–3 days’ exposure, RD mortality risk significantly increased, with women and the elderly being more vulnerable to adverse effects of pollutant exposure. To mitigate these risks, public health efforts should prioritise protecting high-risk populations through targeted interventions, including traffic restrictions near medical facilities during peak pollution periods, community health education on exposure avoidance, and localised emission controls to reduce pollutant dispersion.

## Additional material


Online Supplementary Document
